# The Complete Mitochondrial Genome and Novel Gene Arrangement of the Unique-Headed Bug *Stenopirates* sp. (Hemiptera: Enicocephalidae)

**DOI:** 10.1371/journal.pone.0029419

**Published:** 2012-01-03

**Authors:** Hu Li, Hui Liu, Aimin Shi, Pavel Štys, Xuguo Zhou, Wanzhi Cai

**Affiliations:** 1 Department of Entomology, China Agricultural University, Beijing, China; 2 Entomological Laboratory, Graduate School of Bioresource and Bioenvironmental Sciences, Kyushu University, Fukuoka, Japan; 3 Department of Zoology, Faculty of Science, Charles University, Praha, Czech Republic; 4 Department of Entomology, University of Kentucky, Lexington, Kentucky, United States of America; California State University Fullerton, United States of America

## Abstract

Many of true bugs are important insect pests to cultivated crops and some are important vectors of human diseases, but few cladistic analyses have addressed relationships among the seven infraorders of Heteroptera. The Enicocephalomorpha and Nepomorpha are consider the basal groups of Heteroptera, but the basal-most lineage remains unresolved. Here we report the mitochondrial genome of the unique-headed bug *Stenopirates* sp., the first mitochondrial genome sequenced from Enicocephalomorpha. The *Stenopirates* sp. mitochondrial genome is a typical circular DNA molecule of 15, 384 bp in length, and contains 37 genes and a large non-coding fragment. The gene order differs substantially from other known insect mitochondrial genomes, with rearrangements of both tRNA genes and protein-coding genes. The overall AT content (82.5%) of *Stenopirates* sp. is the highest among all the known heteropteran mitochondrial genomes. The strand bias is consistent with other true bugs with negative GC-skew and positive AT-skew for the J-strand. The heteropteran mitochondrial *atp8* exhibits the highest evolutionary rate, whereas *cox1* appears to have the lowest rate. Furthermore, a negative correlation was observed between the variation of nucleotide substitutions and the GC content of each protein-coding gene. A microsatellite was identified in the putative control region. Finally, phylogenetic reconstruction suggests that Enicocephalomorpha is the sister group to all the remaining Heteroptera.

## Introduction

Mitochondrial (mt) genome sequences are becoming increasingly important for comprehensive evolutionary and population studies. Mt genome sequences are not only more informative than shorter sequences of individual genes, but also provide sets of genome-level characters, such as the relative position of different genes, RNA secondary structures and modes of control of replication and transcription [1]–[4]. For the past two decades, mtDNA has been widely regarded as the molecular marker of choice for the phylogenetic analysis in metazoans because of its abundance in animal tissues, the small genome size, faster rate of evolution, low or absence of sequence recombination, and evolutionary conserved gene products [5], [6], although the applicability of mt genomes as a marker of deeper divergences or highly divergent lineages is still controversial [7], [8]. It is pertinent that the ideal molecular systematic approach would include both nuclear and organellar DNA such as mtDNA markers [9].

The suborder Heteroptera (true bugs) contains over 40,000 species, and majority of the agriculturally important true bugs are the group of phytophagous species attacking cultivated crops. The haematophagous assassin bug *Triatoma dimidiata*, a representative of Triatominae (a subfamily of Reduviidae), is the most important vector of Chagas disease in humans [10], [11]. Relatively few cladistic analyses have addressed relationships among the seven infraorders of Heteroptera during the past 25 years and the hypotheses on infraordinal relationships conflict on crucial points [12]. For example, what is the basal-most sister-group of the majority of Heteroptera - the Enicocephalomorpha (orthodoxy) or Nepomorpha [13]? Mt genome sequences provide a novel insight into the infraordinal relationships of Heteroptera, although the applicability remains to be elucidated. At present, the complete or nearly complete mt genomes of 32 species of heteropterans are available at NCBI (as of April 15, 2011; Table S1). Among these, 15 belong to Pentatomomorpha, nine belong to Nepomorpha, four belong to Cimicomorpha, two belong to Gerromorpha, and two belong to Leptopodomorpha [11], [14]–[16]. Most of the submitted sequences are typically a small double-stranded circular molecule of 14–18 kb in length and contain 13 protein-coding genes (PCGs), two rRNA genes, 22 tRNA genes and a control region (CR). The control region is mostly AT-rich and fulfils a role in the initiation of replication and transcription [17], [18]. To date, mt genome sequences of Enicocephalomorpha and Dipsocoromorpha have not been reported. This lack of information impedes our ability to trace the evolution of the basal groups of Heteroptera based on mt genomes.

Enicocephalomorpha, or unique-headed bugs, are a relatively small group of true bugs [19]–[21], the only ones that engage in nuptial swarming among Heteroptera. They comprise two families, Aenictopecheidae and Enicocephalidae, which include 22 and 322 valid species, respectively, although hundreds of species remain undescribed [22]. Enicocephalomorpha was at one time placed in the Reduvioidea [23], but is now considered the putative sister group to all remaining Heteroptera [12], [20], [24]–[26].

In this paper, we present the complete mt genome of a representative species from the unique-headed bug, *Stenopirates* sp. This is the first species from the Enicocephalomorpha for which the entire mt genome has been sequenced, and for the first time, we report the rearrangement of protein-coding genes in a Heteroptera mt genome. We also discuss architecture of *Stenopirates* sp. mt genome and analyze the RNA secondary structure across the heteropterans. Finally, results from phylogenomic analysis shed lights on the phylogenetic relationship of Enicocephalomorpha among heteropterans.

## Results and Discussion

### Genome organization

**Figure 1:**
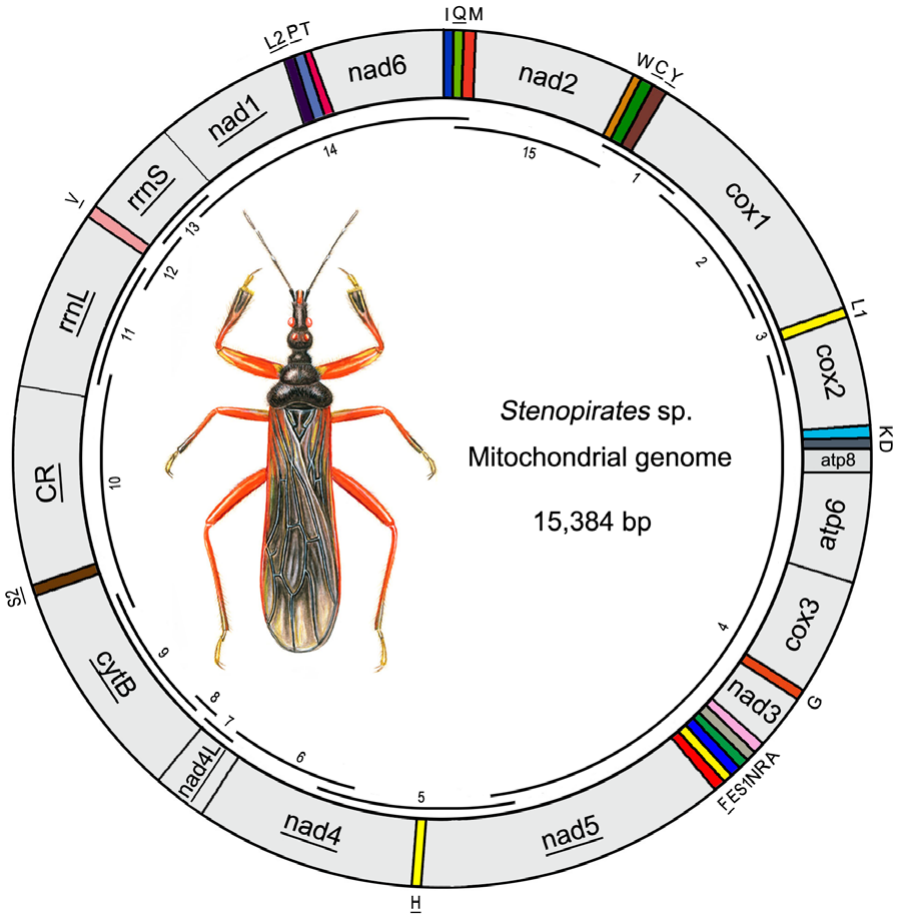
Mitochondrial map of *Stenopirates* sp. The tRNAs are denoted by the color blocks and are labelled according to the IUPACIUB single-letter amino acid codes. Gene name without underline indicates the direction of transcription from left to right, and with underline indicates right to left. Overlapping lines within the circle denote PCR fragments amplified used for cloning and sequencing.

**Figure 2:**
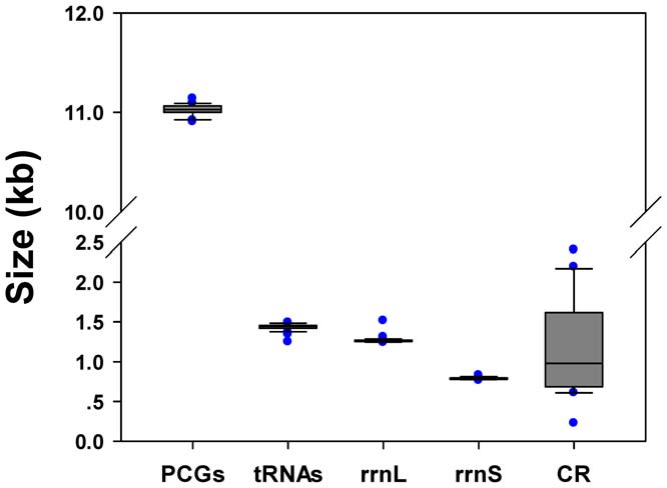
The size of PCGs, *rrnL*, *rrnS*, and CR, respectively, among sequenced true bug mt genomes. Lower horizontal bar, non-outlier smallest observation; lower edge of rectangle, 25 percentile; central bar within rectangle, median; upper edge of rectangle, 75 percentile; upper horizontal bar, non-outlier largest observation; blue circle, outlier.

The complete mt genome of *Stenopirates* sp. is a typical circular DNA molecule of 15, 384 bp in length (GenBank accession no. JN100019; Figure 1). This genome is a medium level of true bug mt genome size, ranging from 14,935 bp to 17,191 bp [14]. Within true bug mt genomes, the length variation was minimal in PCGs, tRNAs, the large and small rRNA subunits (*rrnL* and *rrnS*), but very different in the putative control region (Figure 2; Table S2). The mt genome of *Stenopirates* sp. contains 37 genes in total (13 PCGs, 22 tRNA genes, and two rRNA genes) which are typically present in metazoan mt genomes [17]. Twenty-three genes were transcribed on the majority strand (J-strand), whereas the others were oriented on the minority strand (N-strand). Gene overlaps were found at 17 gene junctions and involved a total of 59 bp; the longest overlap (11 bp) existed between *atp6* and *cox3*. In addition to the large non-coding region, several small non-coding intergenic spacers were present in the *Stenopirates* sp. mt genome and were spread over six positions, ranging in size from 1 to 67 bp (Table S3).

**Figure 3:**
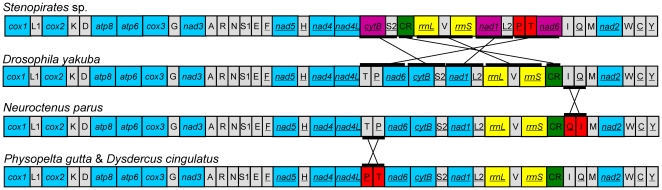
Gene rearrangement of the *Stenopirates* sp. mt genome. Only protein-coding genes (blue), ribosomal RNA genes (yellow) and control region (green) are marked. Blue boxes represent protein-coding genes with the same relative position as in the insect ground pattern, *Drosophila yakuba*; purple boxes and horizontal lines represent gene clusters that changed positions relative to *D. yakuba*; red boxes represent inversions of tRNAs. tRNA genes are abbreviated using the one-letter amino acid code, with L1 = CUN; L2 = UUR; S1 = AGN; S2 = UCN. All genes are transcribed from left to right except those underlined to indicate an opposite transcriptional orientation.

The gene order of the *Stenopirates* sp. mt genome differs largely from those of all other analyzed insect species. Compared to *Drosophila yakuba*, which is considered the representative ground pattern for insect mt genomes [27], 30 of the 38 gene boundaries in *D. yakuba* were conserved in *Stenopirates* sp. The most striking features were the inversion of two tRNA genes (*trnT* and *trnP*) and translocations of five gene clusters (*trnT-trnP-nad6*, *cytB-trnS2*, *nad1-trnL2*, *rrnL-trnV-rrnS* and *CR*) between *nad4L* and *trnI* (Figure 3).

The complete or nearly complete mt genomes of 32 species of Heteroptera have been sequenced and exhibit highly conserved gene order. The mt genomes of three Pentatomomorpha species present gene rearrangements in the inversion of tRNA genes [14]. Two species in the superfamily Pyrrhocoroidea share the same gene order with the inversion of *trnT* and *trnP*. Two tRNA genes (*trnI* and *trnQ*) are inversed in the flat bug *Neuroctenus parus*. Rearrangements of the mt genome are relatively rare events at the evolutionary scale, and, therefore, provide a powerful tool to delimit deep divergences among some metazoan lineages [28]. In comparison to *Stenopirates* sp., rearrangements in other true bugs seem to occur independently. These results suggest that mt gene orders might lack of resolution to deduce phylogenetic relationships among infraorders within Heteroptera, although it has been used extensively to elucidate phylogenetic relations at the superfamily level [29], [30].

### Base composition and codon usage

**Figure 4:**
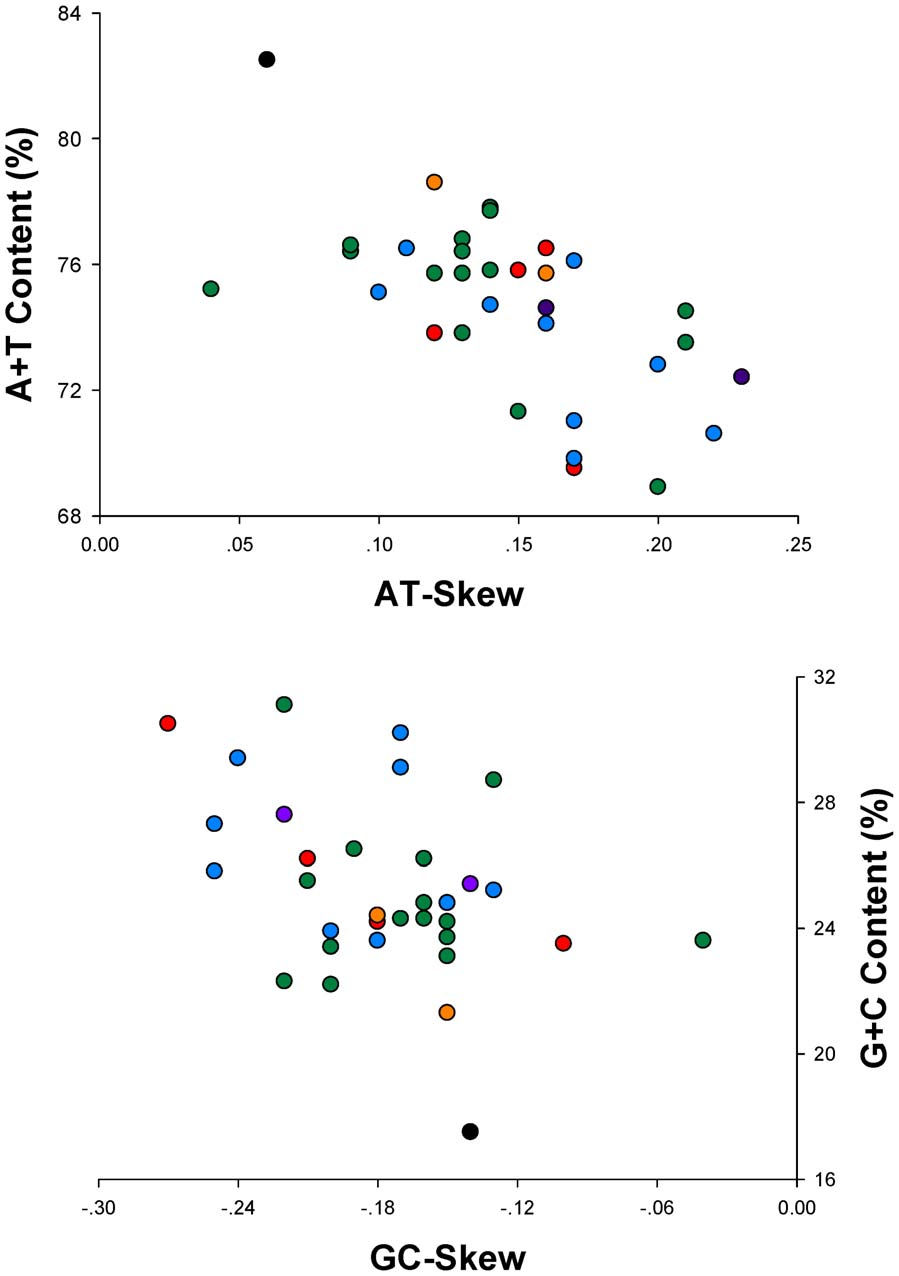
AT% vs AT-Skew and GC% vs GC-Skew in true bug mt genomes. Measured in bp percentage (Y-axis) and level of nucleotide skew (X-axis). Values are calculated on J-strands for full length mt genomes. Green circle, Pentatomomorpha; blue circle, Nepomorpha; red circle, Cimicomorpha; yellow circle, Gerromorpha; purple circle, Leptopodomorpha; black circle, Enicocephalomorpha (*Stenopirates* sp.).

As is the case in other heteropteran mt genome sequences, the nucleotide composition of the *Stenopirates* sp. mt genome was also biased toward A and T (J-strand: A = 43.9%, T = 38.6%, G = 7.5%, C = 10.0%; Table S4). The overall AT content (82.5%) of *Stenopirates* sp. was the highest and much higher than the average AT content of heteropteran mt genomes (Figure 4).

Metazoan mt genomes usually present a clear strand bias in nucleotide composition [31], [32], and the strand bias can be measured as AT- and GC-skews [33]. A comparative analysis of A+T% vs AT-skew and G+C% vs GC-skew across all available mt genomes of true bugs is shown in Figure 4. The average AT-skew of true bug mt genomes was 0.15, ranging from 0.04 in *Hydaropsis longirostris* to 0.23 in *Leptopus* sp., whereas the *Stenopirates* sp. mt genome exhibited a slight AT-skew (0.06) (Table S4). The average GC-skew of true bug mt genomes was −0.18, ranging from −0.04 in *Yemmalysus parallelus* to −0.27 in *Triatoma dimidiata*, and the *Stenopirates* sp. mt genome exhibited a marked GC-skew (−0.14) (Table S4). AT- and GC-skews of true bug mt genomes are consistent compared to the usual strand biases of metazoan mtDNA (positive AT-skew and negative GC-skew for the J-strand).

The reversal of strand asymmetry over the entire mt genome was found to have accelerated gene rearrangement rates [34] and was caused by inversion of replication origin [35]. However, species that have accelerated gene rearrangement rates do not always show a reversal of strand asymmetry, e.g., three *Nasonia* species (Insecta: Hymenoptera) [36], *Thrips imagines* (Insecta: Thysanoptera) [37] and *Stenopirates* sp. in this paper. Therefore, the mechanism of gene rearrangement also needs more in-depth study.

The genome-wide bias toward AT was well documented in the codon usage (Table S5). At the third codon position, A or T were overwhelmingly overrepresented compared to G or C. The overall pattern was very similar among the true bugs, with similar frequency of occurrences of various codons within a single codon family. There was a strong bias toward AT-rich codons with the six most prevalent codons in *Stenopirates* sp., as in order, TTA-Leu (12.76%), ATT-Ile (11.86%), ATA-Met (10.75%), TTT-Phe (9.93%), AAT-Asn (6.72%) and TAT-Tyr (4.49%) (Table S5).

### Protein-coding genes

The total length of all 13 PCGs was 11,056 bp, and accounted for 71.87% of the entire length of *Stenopirates* sp. mt genome. The overall AT content of PCGs was 82.05%, ranging from 74.0% (*cox1*) to 90.4% (*atp8*). Start and stop codons were determined based on alignments with the corresponding genes of other true bugs (Table S3). Five genes (*atp6, cox3, nad4, cytB, nad1*) used the standard ATG start codon, four genes (*nad2, atp8, nad4L, nad6*) started with ATA and three genes (*cox2, nad3, nad5*) initiated with ATT. *Cox1* most likely started with codon TTG. Nine genes employ a complete translation termination codon, either TAG (*nad3, cytB*) or TAA (*nad2, cox1, atp8, atp6, nad4L, nad1, nad6*), whereas the remaining four have incomplete stop codons T. The presence of an incomplete stop codon is common in metazoan mt genomes [17] and these truncated stop codons are presumed to be completed via post-transcriptional polyadenylation [38].

**Figure 5:**
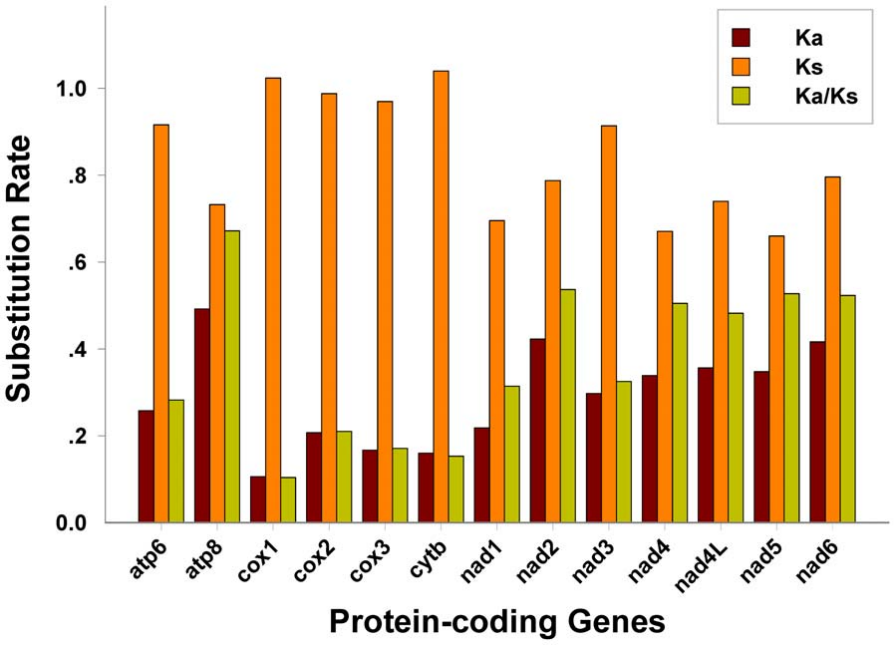
Evolutionary rates of true bug mt genomes. The rate of non-synonymous substitutions (Ka), the rate of synonymous substitutions (Ks) and the ratio of the rate of non-synonymous substitutions to the rate of synonymous substitutions (Ka/Ks) for each PCG.

The rate of non-synonymous substitutions (Ka), the rate of synonymous substitutions (Ks), and the ratio of Ka/Ks were calculated for each PCG, respectively. In this respect, *atp8* showed the highest evolutionary rates, followed by *nad2*, while *cox1* appeared to be the lowest (Figure 5). Notably, the ratio of Ka/Ks for each and every PCG was below 1, indicating that these genes are evolving under the purifying selection [39], [40]. Furthermore, a negative correlation was observed between the Ka/Ks and the GC content of each PCG (R = −0.916, *P*<0.01) (Table S6), which indicate that the variation of GC content probably causes the different evolutionary patterns among genes [14].

### Transfer RNAs

**Figure 6:**
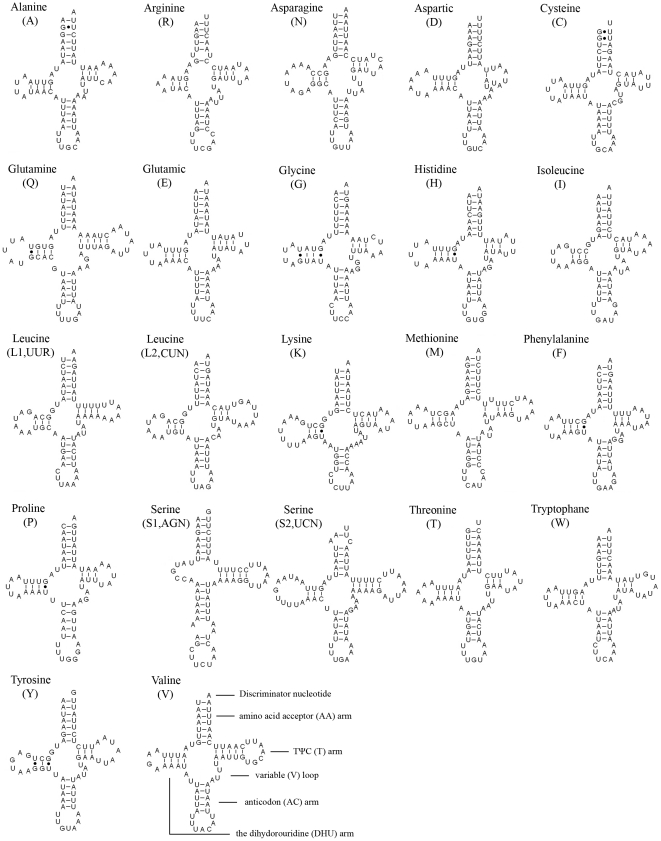
Inferred secondary structure of 22 tRNAs of *Stenopirates* sp. The tRNAs are labeled with the abbreviations of their corresponding amino acids. Inferred Watson-Crick bonds are illustrated by lines, whereas GU bonds are illustrated by dots.

The entire complement of 22 tRNAs typical of arthropod mt genomes was found in *Stenopirates* sp. and schematic drawings of their respective secondary structures are shown in Figure 6. Most of the tRNAs could be folded as classic clover-leaf structures, with the exception of *trnS1*, in which its DHU arm simply formed a loop. This phenomenon is a common theme in the true bug mt genomes. The aberrant tRNAs possess non-Watson-Crick matches, aberrant loops, or extremely short arms are common in metazoan mt genomes [17]. Whether or not the aberrant tRNAs lose their respective functions is still unknown, however, a post-transcriptional RNA editing mechanism has been proposed to sustain functions for these modified tRNAs [41], [42].

### Ribosomal RNAs

The ends of rRNA genes are impossible to be precisely determined by DNA sequencing alone, so they are assumed to extend to the boundaries of flanking genes [43], [44]. The *srRNA* was assumed to fill up the blanks between *tRNA-V* and *nad1*. For the boundary between the *lrRNA* gene and the non-coding putative control region, alignments with homologous sequences in other heteropteran mt genomes were applied to determine the 3′-end of the gene [11], [14]–[16]. The length of *rrnL* and *rrnS* of *Stenopirates* sp. was determined to be 1, 245 bp and 829 bp, respectively.

**Figure 7:**
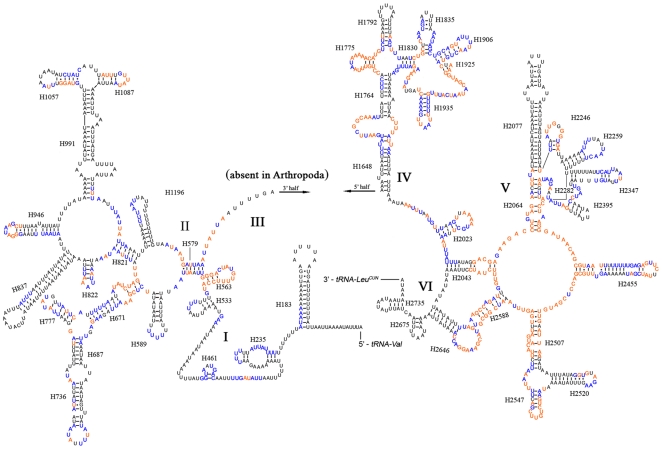
Predicted secondary structure of the *rrnL* gene in *Stenopirates* sp. The nucleotides showing 100% identities among true bugs are marked with orange color, and more than or equal to 75% identities are marked with blue color. Roman numerals denote the conserved domain structure. Inferred Watson-Crick bonds are illustrated by lines, GU bonds by dots.

**Figure 8:**
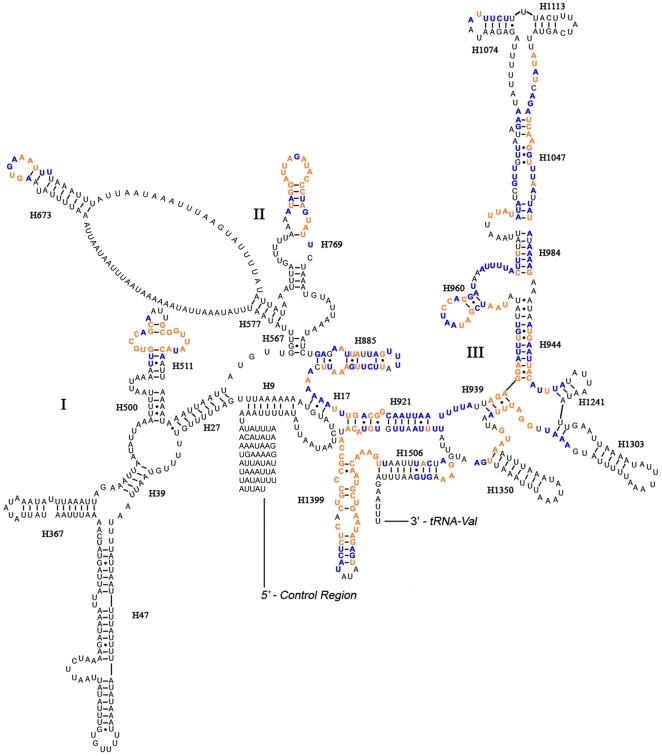
Predicted secondary structure of the *rrnS* gene in *Stenopirates* sp. The nucleotides showing 100% identities among true bugs are marked with orange color, and ≥75% identities are marked with blue color. Roman numerals denote the conserved domain structure. Inferred Watson-Crick bonds are illustrated by lines, GU bonds by dots.

Both *rrnL* and *rrnS* are incongruent with the secondary structure models proposed for other insects [45]–[48]. The secondary structure of *Stenopirates* sp. *rrnL* consisted of six structural domains (domain III is absent in arthropods) (Figure 7). Among sequenced true bugs, the sequence variations were too high in some regions for meaningful structural comparisons. Overall, the 5′ and 3′ ends, some helices (H183, H589, H687, H736, H837, H991, H1196, H1648, H1792, H2077, H2520), and domain VI were the most variable regions. Domains IV and V were more conserved. The secondary structure of *rrnS* contained three domains (Figure 8). Conservative sites were mainly in domain III and some helices (loops of H673, H769 and H889) in domain II.

### Non-coding regions

The largest non-coding region (765 bp) was flanked by *trnS2* and *rrnL* in the *Stenopirates* sp. mt genome. It was highly enriched in AT (74.9%) and could form stable stem-loop secondary structures. Based on these features, it possibly functions as a control region [17], [49].

**Figure 9:**
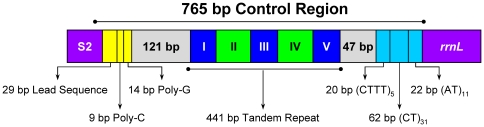
Organization of the mitochondrial control region of *Stenopirates* sp. The control region flanking genes *trnS2* (S2) and *rrnL* are represented in purple boxes. The blue and green boxes with roman numerals indicate the tandem repeat region; grey boxes represent the stem-loop region.

Based on the sequence pattern, the control region could be subdivided into five parts (Figure 9). The first region (10,779–10,807) was a 29 bp leading sequence enriched in AT. The second region (10,808–10,830) included the 9 bp poly-C and 14 bp poly-G. The poly-G has been reported in assassin bug *Agriosphodrus dohrni* (referred as G element) [50], and triatomine bugs *Rhodnius prolixus* and *Triatoma dimidiata* (referred as Gs) [11], and some dipterans (referred as G islands) [48]. The possible involvement of this unique motif in insect replication and transcription initiation is one topic for the future research [51]–[53]. The third region (10,952–11,392) contained five (I–V) tandem repeats including two (I & III) 80 bp, one (V) 52 bp (a partial copy of the anterior repeat unit), and two (II & IV) repeats (with substitute of few nucleotides). The maximum size difference found in the control regions across all sequenced true bug mt genomes was 2,756 bp, indicating that strong size variation among true bug mt genomes is significantly correlated to the control regions (Figure 2). This result is consistent with previous findings from other insects [14], [51], [54]. In fact, the control region has been identified as the source of size variation in the entire mt genome, usually due to the presence of a variable copy number of repetitive elements [49]. Repeated sequences are common in the control region for most insects, and length variations due to the various numbers of repeats are not without precedent [11]. Consequently, analysis of the repeat units among individuals from different geographical locations may shed light on the geographical structuring and phylogenetic relationships of species. The fact that tandem repeats are non-conserved among these heteropteran mt genomes indicates a lack of a functional role. Replication slippage is regarded as a dominant mechanism to account for the existence of tandem repeats [55], [56].

**Figure 10:**
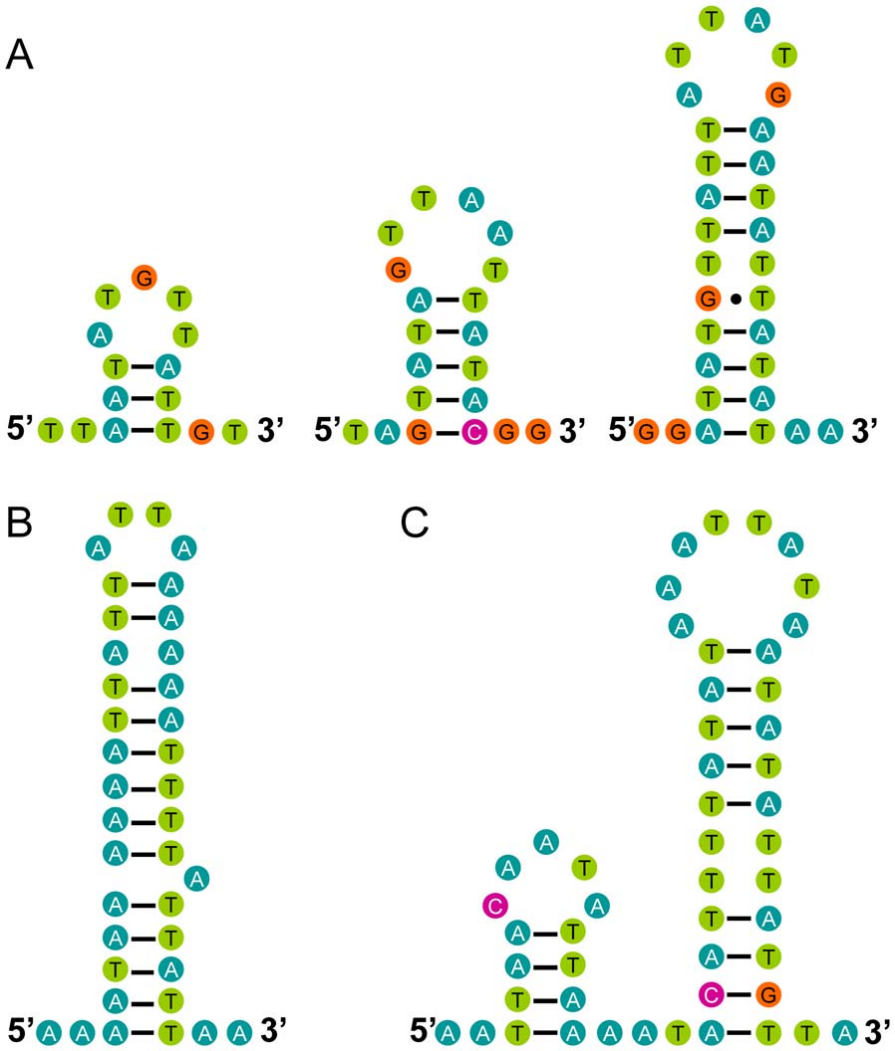
Secondary structures of non-coding regions of the mt genome of *Stenopirates* sp. Secondary structure of non-coding regions between (A) *trnS2* and *rrnL* (CR); (B) *cytB* and *trnS2;* (C) *trnT* and *nad6*. Inferred Watson-Crick bonds are illustrated by lines, GU bonds by dots.

The fourth region (10,831–10,951 & 11,393–11,439) was near the tandem repeat region, and stem-loop structures which may be involved in the initiation of the replication of animal mtDNA [57] could be folded (Figure 10A), but none of these structures had flanking sequences similar to those that are conserved in the control region of the mt genomes of insects [58]. The fifth region (11,440–11,543) contained five CTTT-repeats, 31 CT-repeats and 22 AT- repeats. This domain can be considered a microsatellite [59]. In arthropod mtDNA such microsatellites are rare and only been reported for butterflies [47], the Asian arowana, *Scleropages formosus* [60], and a house dust mite, *Dermatophagoides pteronyssinus* [58]. This is remarkable because a mt microsatellite has not been reported for any heteropteran species. As described previously, four other stretches of non-coding nucleotides were found outside the control region. These short sequences can fold into stable stem-loop structures (Figure 10B & C) which may function as splicing recognition sites during processing of the transcripts [61].

### Phylogenetic analysis

**Figure 11:**
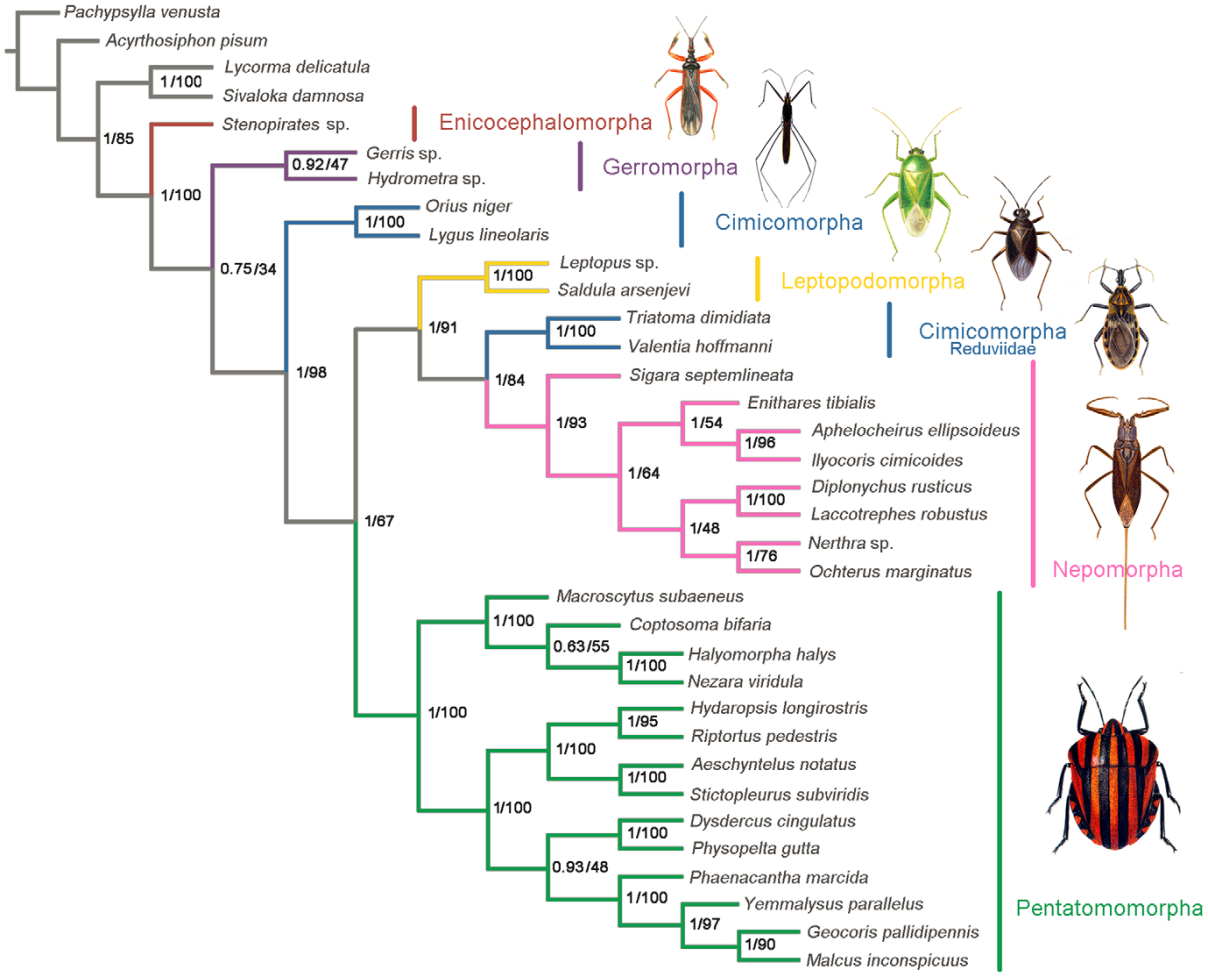
Phylogenetic relationships among the sequenced true bugs. Phylogenetic analyses were carried out for the 31 true bugs based on all 13 protein-coding genes from their respective mt genomes. The tree was rooted with four outgroups (*P. venusta*, *A. pisum*, *S. damnosa* and *L. delicatula*). Numbers at the nodes are Bayesian posterior probabilities (left) and ML bootstrap values (right).

Phylogenetic analyses were carried out using nucleotide sequences of 13 mt PCGs from 31 heteropteran species and 4 outgroup hemipteran insect species (*Pachypsylla venusta* [30], *Acyrthosiphon pisum* [62], *Sivaloka damnosa* [63] and *Lycorma delicatula* [16]). BI and ML analyses generated identical tree topologies (Figure 11).

The seven-infraorder classification of Heteroptera has been accepted by most researchers [20], [26], however phylogenetic relationships among infraorders are still controversial [13], [16], [20], [26], [64]–[66]. The major problem is the basalmost sister-group of the majority of Heteroptera, the Enicocephalomorpha (orthodoxy) or Nepomorpha [13].

In the present study, the sister-relationship within the individual infraorders (as shown in Figure 11) are supported for the Pentatomomorpha (14 taxa), Nepomorpha (8 taxa), Leptopodomorpha (2 taxa) and Gerromorpha (2 taxa) by BI and ML analyses. In addition, both ML and BI analyses are highly supportive of the contention that *Stenopirates* sp. (Enicocephalomorpha) is the sister group to all the remaining Heteroptera [26], [67].

Within Cimicomorpha, Reduviidae was paraphyletic with respect to Anthocoridae and Miridae in our trees, and this is largely incongruent with previous phylogenetic works [65], [68], [69]. The mt genome data in this study, however, may be limited to resolve the phylogeny of Cimicomorpha, and increased taxon sampling will be required to resolve this problem.

The ability of mt genome data to resolve infraordinal relationships of Heteroptera has not been fully evaluated. This study provides the initial evidence for the feasibility of using mt genome data to resolve infraordinal relationships of Heteroptera; however, the prerequisite is to ensure the integrity and representative of the infraorder-level taxa.

Future directions should focus on the following problems raised in the modern literature: (a) Are Dipsocoromorpha monophyletic and sister to the rest of Heteroptera (orthodoxy) or are they formed by two distinct clades with uncertain relationships (Štys, *in prep.*)? (b) Are Nepomorpha monophyletic (orthodoxy) or should the Pleomorpha be excluded and its origin seeked for elsewhere [16]? (c) Are some “Thaumastocoridae” pentatomomorphans and others cimicomorphans [65]? (d) Are the Pentatomomorpha monophyletic (orthodoxy) or should the Aradimorpha be excluded and its origin be seeked elsewhere [66]? and (e) What is the mutual relationship of Nepomorpha (*s. lat.*), Leptopodomorpha, and the truly terrestrial true bugs?

### Summary

This is the first description of the complete mt genome of a species belonging to Enicocephalomorpha, an infraorder within Heteroptera. The overall AT content of *Stenopirates* sp. is the highest among sequenced heteropteran mt genomes. Although the gene order of the *Stenopirates* sp. mt genome is extremely rearranged and represents a new pattern, rearrangements exhibit relatively rare events and seem to occur independently within true bug mt genomes. Gene order comparison indicated that mt gene order seems less useful for deduction of phylogenetic relationships among infraorders of Heteroptera. Comparative analyses suggest that the gene size, gene content, and base composition are comparatively conserved among true bug mt genomes. PCGs exhibit a different nucleotide substitution pattern, negatively correlated with GC content. True bugs mt *atp8* represents the highest evolutionary rate; whereas *cox1* appears to be the lowest. Most of the tRNAs can be folded as classic clover-leaf structures, with the exception of *trnS1*, in which its DHU arm simply forms a loop. In addition to stem-loop structures in the control region, another common feature is the existence of tandem repeats. Phylogenetic analysis using concatenated PCG sequences succeeded in corroborating hypothesis on sister-group relationship of Enicocephalomorpha to other heteropterans. The present study demonstrates the great effectiveness of mt genome for inferring phylogenetic relationships at the infraorder level.

## Materials and Methods

### Ethics statement

No specific permits were required for the insect collected for this study in Taiwan. The insect specimens were collected on the road side by sweeping. The field studies did not involve endangered or protected species. The species in the genus of Stenopirates are common small insects and are not included in the “List of Protected Animals in China”.

### Samples and DNA extraction

The Oriental and East Palaearctic genus *Stenopirates* (Enicocephalinae: Enicocephalini) includes 8 described and about 20 undescribed species [19]. *Stenopirates* sp. adult males were collected from Pingdong, Taiwan, China, in May 2009. All collections were initially preserved in 95% ethanol in the field, and transferred to −20°C for the long-term storage upon the arrival at the China Agricultural University (CAU). The genomic DNA was extracted from muscle tissues of a single *Stenopirates* sp. 's thorax using a CTAB-based protocol [70].

### PCR amplification and sequencing

The mt genome of *Stenopirates* sp. was generated by amplification of overlapping PCR fragments (Figure 1 and Table S7). Initially, eleven fragments were amplified using the universal primer sets [71]. Four perfectly matched primers (Table S7) were designed based on the read of these short fragments for the secondary PCRs.

Short PCRs (<1.5 kb) were carried out using Qiagen Taq DNA polymerase (Qiagen, Beijing, China) with the following cycling conditions: 5 min at 94°C, followed by 35 cycles of 50 s at 94°C, 50 s at 48–55°C, 1–2 min at 72°C depending on the size of amplicons, and the subsequent final elongation step at 72°C for 10 min. Long PCRs (>1.5 kb) were performed using NEB Long Taq DNA polymerase (New England BioLabs, Ipswich, MA) under the following cycling conditions: 30 s at 95°C, followed by 40 cycles of 10 s at 95°C, 50 s at 48–55°C, 3–6 min at 68°C depending on the size of amplicons, and the final elongation step at 68°C for 10 min . The quality of PCR products were evaluated by spectrophotometry and agarose gel electrophoresis.

The PCR fragments were ligated into the pGEM-T Easy Vector (Promega) and resulting plasmid DNAs were isolated using the TIANprp Midi Plasmid Kit (Qiagen, Beijing, China). All fragments were sequenced in both directions using the BigDye Terminator Sequencing Kit (Applied Bio Systems) and the ABI 3730XL Genetic Analyzer (PE Applied Biosystems, San Francisco, CA, USA) with two vector-specific primers and internal primers for primer walking.

### Annotation and bioinformatic analysis

The complete mt genome of *Stenopirates* sp. has been deposited in GenBank under accession number JN100019. Mt DNA sequences were proof-read and aligned into contigs in BioEdit v.7.0.5.3 [72]. PCGs and rRNA genes were identified based on sequence similarity with published insect mt sequences from public domains (e.g., GenBank).

The tRNA genes were identified by tRNAscan-SE Search Server v.1.21 [73] with default settings. Some tRNA genes that could not be determined by tRNAscan-SE were determined in the unannotated regions by sequence similarity to tRNAs of other heteropterans. The base composition, codon usage, and nucleotide substitution were analyzed with Mega 4.0 [74].

The software packages DnaSP 5.0 [75] was used to calculate the number of synonymous substitutions per synonymous site (Ks) and the number of nonsynonymous substitutions per nonsynonymous site (Ka) for each PCG.

### Construction of secondary structures of rRNAs and non-coding Regions

Secondary structures of the small and large subunits of rRNAs were inferred using models predicted for *Drosophila* [45], *Apis mellifera* [46], *Manduca sexta* [47] and *Ruspolia dubia* [48]. Stem-loops were named according to the convention of [46], as well as [47]. Regions lacking significant homology and other non-coding regions were folded using Mfold [76].

### Phylogenetic analysis

Phylogenetic analyses were performed based on the 31 complete or nearly complete mt genomes of true bugs from GenBank (Table S1). Two species from Sternorrhyncha and two species from “Auchenorrhyncha”: Fulgoromorpha were selected as outgroups. Based on an analysis of mt genomes of nine Nepomorpha and five other hemipterans, Pleidae were recovered as the sister group to Geocorisae + Leptopodomorpha + remaining Nepomorpha, and were suggested to be raised from a superfamily to the infraorder Plemorpha [13]. Similarly, the phylogenetic position of “Aradoidea” or “Aradimorpha” was also the problem [68]. Since we didn't add samples to solve these problems, *Paraplea frontalis* (Nepomorpha: Pleidae) and *Neuroctenus parus* (Pentatomomorpha: Aradidae) were treated as *incertae sedis*. These two species were not included in the phylogenetic analyses to ensure the stability of the topology.

DNA alignment was inferred from the amino acid alignment of 13 PCGs using Clustal X [77] as implemented in the Mega 4.0 [74], which can translate between DNA and amino acid sequences within alignments. Alignments of individual genes were then concatenated excluding the stop codon. Model selection was based on Modeltest 3.7 [78] for nucleotide sequences. According to the Akaike information criterion, the GTR+I+G model was optimal for analysis with nucleotide alignments. MrBayes v.3.1.2 [79] and a PHYML online web server [80], [81] were employed to reconstruct the phylogenetic trees. In Bayesian inference, two simultaneous runs of 3,000,000 generations were conducted. Each set was sampled every 200 generations with a burnin of 25% [16], [54], [82], [83]. Trees inferred prior to stationarity were discarded as burnin, and the remaining trees were used to construct a 50% majority-rule consensus tree. In the ML analysis, the parameters were estimated during analysis and the node support values were assessed by bootstrap re-sampling (BP) [84] calculated using 100 replicates.

## Supporting Information

Table S1 **Summary of taxonomic groups used in this study.**
(DOCX)

Table S2 **The Size of PCGs, tRNAs, rrnL, rrnS, and CR, respectively, among sequenced true bug mt genomes.**
(DOCX)

Table S3 **Organization of** ***Stenopirates*** **sp. mt genome.**
(DOCX)

Table S4 **Base composition and strand bias in true bug mt genomes.**
(DOCX)

Table S5 **Codon usage of protein-coding genes in the** ***Stenopirates*** **sp. mt genome.**
(DOCX)

Table S6 **Different evolutionary patterns among protein-coding genes.**
(DOCX)

Table S7 **Primer sequences used in this study.**
(DOCX)
