# Supplementary material for: The Complete Mitochondrial Genome and Novel Gene Arrangement of the Unique-Headed Bug Stenopirates sp. (Hemiptera: Enicocephalidae)
Source: PLoS One. 2012 Jan 3;7(1):e29419. doi: 10.1371/journal.pone.0029419 (PMC3250431; doi:10.1371/journal.pone.0029419)
Supplement: Table S1 — Summary of taxonomic groups used in this study. (DOCX) [file pone.0029419.s001.docx]

**Table S1. Summary of taxonomic groups used in this study**

| **Order/suborder** | **Infraorder/**  **superfamily** | **Family** | **Species** | **Accession Number** |
| --- | --- | --- | --- | --- |
| Sternorrhyncha |  |  |  |  |
|  | Psyllomorpha |  |  |  |
|  | Psylloidea | Psyllidae | *Pachypsylla venusta* (Osten-Sacken) | NC_006157 |
|  | Aphidomorpha |  |  |  |
|  | Aphidoidea | Aphididae | *Acyrthosiphon pisum* (Harris) | NC_011594 |
| Auchenorrhyncha |  |  |  |  |
|  | Fulgoromorpha |  |  |  |
|  | Fulgoroidea | Fulgoridae | *Lycorma delicatula* (White) | NC_012835 |
|  |  | Issidae | *Sivaloka damnosus* Chou et Lu | NC_014286 |
| Heteroptera |  |  |  |  |
|  | Enicocephalomorpha |  |  |  |
|  |  | Enicocephalidae | *Stenopirates* sp. | JN100019 |
|  | Gerromorpha |  |  |  |
|  | Hydrometroidea | Hydrometridae | *Hydrometra* sp. | NC_012842 |
|  | Gerroidea | Gerridae | *Gerris* sp. | NC_012841 |
|  | Nepomorpha |  |  |  |
|  | Corixoidea | Corixidae | *Sigara septemlineata*  (Paiva) | FJ456941 |
|  | Ochteroidea | Gelastocoridae | *Nerthra* sp. | [NC_012838](http://www.ncbi.nlm.nih.gov/nuccore/NC_012838) |
|  |  | Ochteridae | *Ochterus marginatus* (Latreille) | NC_012820* |
|  | Notonectoidea | Notonectidae | *Enithares tibialis* Liu et Zheng | NC_012819 |
|  |  | Pleidae | *Paraplea frontalis* (Fieber) | NC_012822 |
|  | Nepoidea | Nepidae | *Laccotrephes robustus* Stål | NC_012817 |
|  |  | Belostomatidae | *Diplonychus rusticus* (Fabricius) | FJ456939* |
|  | Naucoroidea | Naucoridae | *Ilyocoris cimicoides* (Linnaeus) | NC_012845 |
|  |  | Aphelocheiridae | *Aphelocheirus ellipsoideus* Liu et Ding | FJ456940* |
|  | Leptopodomorpha |  |  |  |
|  | Saldoidea | Saldidae | *Saldula arsenjevi* (Vinokurov) | NC_012463 |
|  | Leptopodoidea | Leptopodidae | *Leptopus* sp. | FJ456946 |
|  | Cimicomorpha |  |  |  |
|  | Cimicoidea | Anthocoridae | *Orius niger* Wolff | NC_012429* |
|  | Reduvioidea | Reduviidae | *Triatoma dimidiata* (Latreille) | NC_002609 |
|  |  |  | *Valentia hoffmanni* China | NC_012823 |
|  | Miroidea | Miridae | *Lygus lineolaris* (Palisot de Beauvois) | EU401991* |
|  | Pentatomomorpha |  |  |  |
|  | Aradoidea | Aradidae | *Neuroctenus parus* Hsiao | NC_012459 |
|  | Pentatomoidea | Pentatomidae | *Nezara viridula* (Linnaeus) | NC_011755 |
|  |  |  | *Halyomorpha halys* (Stål) | NC_013272 |
|  |  | Cydnidae | *Macroscytus subaeneus* (Dallsa) | NC_012457* |
|  |  | Plataspidae | *Coptosoma bifaria* Montandon | NC_012449 |
|  | Lygaeoidea | Berytidae | *Yemmalysus parallelus* Stusak | NC_012464 |
|  |  | Colobathristidae | *Phaenacantha marcida* Horvath | NC_012460* |
|  |  | Malcidae | *Malcus inconspicuus* Štys | NC_012458 |
|  |  | Geocoridae | *Geocoris pallidipennis* (Costa) | NC_012424* |
|  | Pyrrhocoroidea | Largidae | *Physopelta gutta* (Burmeister) | NC_012432 |
|  |  | Pyrrhocoridae | *Dysdercus cingulatus* (Fabricius) | NC_012421 |
|  | Coreoidea | Alydidae | *Riptortus pedestris* Fabricius | NC_012462 |
|  |  | Coreidae | *Hydaropsis longirostris* (Hsiao) | NC_012456 |
|  |  | Rhopalidae | *Aeschyntelus notatus* Hsiao | NC_012446* |
|  |  |  | *Stictopleurus subviridis* Hsiao | NC_012888 |

“*”:Incomplete mt genome.
