# Supplementary material for: The Complete Mitochondrial Genome and Novel Gene Arrangement of the Unique-Headed Bug Stenopirates sp. (Hemiptera: Enicocephalidae)
Source: PLoS One. 2012 Jan 3;7(1):e29419. doi: 10.1371/journal.pone.0029419 (PMC3250431; doi:10.1371/journal.pone.0029419)
Supplement: Table S2 — The Size of PCGs, tRNAs, rrnL, rrnS, and CR, respectively, among sequenced true bug mt genomes. (DOCX) [file pone.0029419.s002.docx]

**Table S2. The Size of PCGs, tRNAs, rrnL, rrnS, and CR, respectively, among sequenced true bug mt genomes**

| **Species** | **PCGs** | **tRNAs** | **rrnL** | **rrnS** | **CR** |
| --- | --- | --- | --- | --- | --- |
| *Aeschyntelus notatus* | 11061.00 | 1454.00 | 1256.00 | 784.00 | - |
| *Aphelocheirus ellipsoideus* | 11013.00 | 1453.00 | 1269.00 | 782.00 | - |
| *Coptosoma bifaria* | 11019.00 | 1458.00 | 1280.00 | 803.00 | 1585.00 |
| *Diplonychus rusticus* | 10992.00 | 1461.00 | 1268.00 | 797.00 | 1617.00 |
| *Dysdercus cingulatus* | 11079.00 | 1440.00 | 1259.00 | 792.00 | - |
| *Enithares tibialis* | 11091.00 | 1483.00 | 1258.00 | 784.00 | 646.00 |
| *Geocoris pallidipennis* | 11034.00 | 1391.00 | 1241.00 | 800.00 | 781.00 |
| *Gerris* sp. | 10941.00 | 1442.00 | 1255.00 | 795.00 | - |
| *Halyomorpha halys* | 11013.00 | 1492.00 | 1291.00 | 813.00 | 1810.00 |
| *Hydaropsis longirostris* | 11022.00 | 1455.00 | 1251.00 | 779.00 | 1991.00 |
| *Hydrometra* sp. | 10983.00 | 1431.00 | 1258.00 | 786.00 | 694.00 |
| *Ilyocoris cimicoides* | 11085.00 | 1381.00 | 1256.00 | 783.00 | 609.00 |
| *Laccotrephes robustus* | 11028.00 | 1443.00 | 1254.00 | 773.00 | - |
| *Leptopus* sp. | 11139.00 | 1246.00 | 1248.00 | - | - |
| *Lygus lineolaris* | 11073.00 | 1436.00 | 1256.00 | 788.00 | - |
| *Macroscytus subaeneus* | 10917.00 | 1342.00 | 1256.00 | 781.00 | 1180.00 |
| *Malcus inconspicuus* | 11058.00 | 1397.00 | 1309.00 | 779.00 | - |
| *Nerthra* sp. | 11094.00 | 1481.00 | 1239.00 | 786.00 | 1450.00 |
| *Neuroctenus parus* | 10917.00 | 1441.00 | 1514.00 | 785.00 | 649.00 |
| *Nezara viridula* | 11022.00 | 1486.00 | 1283.00 | 814.00 | 2190.00 |
| *Ochterus marginatus* | 11082.00 | 1456.00 | 1254.00 | 778.00 | - |
| *Orius niger* | 10986.00 | 1458.00 | 1256.00 | 787.00 | - |
| *Paraplea frontalis* | 11043.00 | 1439.00 | 1238.00 | 790.00 | 608.00 |
| *Phaenacantha marcida* | 10929.00 | 1451.00 | 1262.00 | 779.00 | 224.00 |
| *Physopelta gutta* | 10905.00 | 1404.00 | 1270.00 | 826.00 | - |
| *Riptortus pedestris* | 11028.00 | 1446.00 | 1260.00 | 785.00 | 2400.00 |
| *Saldula arsenjevi* | 11091.00 | 1367.00 | 1255.00 | 764.00 | 687.00 |
| *Sigara septemlineata* | 11019.00 | 1442.00 | 1247.00 | 772.00 | 1207.00 |
| *Stenopirates jeanneli* | 11056.00 | 1425.00 | 1245.00 | 829.00 | 765.00 |
| *Stictopleurus subviridis* | 11037.00 | 1453.00 | 1263.00 | 776.00 | 685.00 |
| *Triatoma dimidiata* | 11064.00 | 1454.00 | 1270.00 | 781.00 | 2160.00 |
| *Valentia hoffmanni* | 11058.00 | 1450.00 | 1256.00 | 777.00 | 724.00 |
| *Yemmalysus parallelus* | 11004.00 | 1378.00 | 1257.00 | 796.00 | 1181.00 |
| Avg. | 11026.76 | 1431.39 | 1267.70 | 788.88 | 1174.68 |

“-”: Data are currently not available.
