# Supplementary material for: The Complete Mitochondrial Genome and Novel Gene Arrangement of the Unique-Headed Bug Stenopirates sp. (Hemiptera: Enicocephalidae)
Source: PLoS One. 2012 Jan 3;7(1):e29419. doi: 10.1371/journal.pone.0029419 (PMC3250431; doi:10.1371/journal.pone.0029419)
Supplement: Table S3 — Organization of Stenopirates sp. mt genome. (DOCX) [file pone.0029419.s003.docx]

**Table S3. Organization of *Stenopirates* sp.** **mt genome**

| **Gene** | **Direction** | **Location (bp)** | **Size (bp)** | **Anticodon** | **Start Codon** | **Stop Codon** | **Intergenic**  **Nucleotide^*^** |
| --- | --- | --- | --- | --- | --- | --- | --- |
| *trnI* | F | 1-63 | 63 | 30-32 GAT |  |  | -2 |
| *trnQ* | R | 61-129 | 69 | 97-99 TTG |  |  | -3 |
| *trnM* | F | 129-197 | 69 | 160-162 CAT |  |  | 0 |
| *nad2* | F | 198-1184 | 987 |  | ATA | TAA | 0 |
| *trnW* | F | 1183-1247 | 65 | 1213-1215 TCA |  |  | -2 |
| *trnC* | R | 1257-1318 | 62 | 1286-1288 GCA |  |  | 9 |
| *trnY* | R | 1318-1381 | 64 | 1350-1352 GTA |  |  | -1 |
| *cox1* | F | 1381-2919 | 1539 |  | TTG | TAA | -1 |
| *trnL1* | F | 2915-2979 | 65 | 2944-2946 TAA |  |  | -5 |
| *cox2* | F | 2980-3649 | 685 |  | ATT | T- | 0 |
| *trnK* | F | 3650-3717 | 68 | 3682-3684 CTT |  |  | 0 |
| *trnD* | F | 3716-3776 | 61 | 3747-3749 GTC |  |  | -2 |
| *atp8* | F | 3777-3932 | 156 |  | ATA | TAA | 0 |
| *atp6* | F | 3926-4606 | 681 |  | ATG | TAA | -7 |
| *cox3* | F | 4596-5379 | 784 |  | ATG | T- | -11 |
| *trnG* | F | 5380-5440 | 61 | 5409-5411 TCC |  |  | 0 |
| *nad3* | F | 5441-5794 | 354 |  | ATT | TAG | 0 |
| *trnA* | F | 5793-5854 | 62 | 5822-5824 TGC |  |  | -2 |
| *trnR* | F | 5854-5914 | 61 | 5880-5882 TCG |  |  | -1 |
| *trnN* | F | 5914-5977 | 64 | 5945-5947 GTT |  |  | -1 |
| *trnS1* | F | 5977-6043 | 67 | 6002-6004 TCT |  |  | -1 |
| *trnE* | F | 6043-6105 | 63 | 6073-6075 TTC |  |  | -1 |
| *trnF* | R | 6104-6167 | 64 | 6135-6137 GAA |  |  | -2 |
| *nad5* | R | 6168-7866 | 1699 |  | ATT | T- | 0 |
| *trnH* | R | 7867-7928 | 62 | 7896-7898 GTG |  |  | 0 |
| *nad4* | R | 7929-9258 | 1330 |  | ATG | T- | 0 |
| *nad4L* | R | 9252-9530 | 279 |  | ATA | TAA | -7 |
| *cytB* | F | 9578-10714 | 1137 |  | ATG | TAG | 47 |
| *trnS2* | F | 10705-10778 | 74 | 10739-10741 TGA |  |  | -10 |
| *CR* |  | 10779-11543 | 765 |  |  |  | 0 |
| *rrnL* | R | 11544-12788 | 1245 |  |  |  | 0 |
| *trnV* | R | 12789-12857 | 69 | 12825-12827 TAC |  |  | 0 |
| *rrnS* | R | 12858-13686 | 829 |  |  |  | 0 |
| *nad1* | R | 13687-14616 | 930 |  | ATG | TAA | 0 |
| *trnL2* | R | 14618-14683 | 66 | 14652-14654 TAG |  |  | 1 |
| *trnP* | R | 14686-14748 | 63 | 14716-14718 TGG |  |  | 2 |
| *trnT* | F | 14816-14878 | 63 | 14846-14848 TGT |  |  | 67 |
| *nad6* | F | 14892-2 | 495 |  | ATA | TAA | 13 |

“*”: Negative numbers indicate that adjacent genes overlap.
