# Supplementary material for: The Complete Mitochondrial Genome and Novel Gene Arrangement of the Unique-Headed Bug Stenopirates sp. (Hemiptera: Enicocephalidae)
Source: PLoS One. 2012 Jan 3;7(1):e29419. doi: 10.1371/journal.pone.0029419 (PMC3250431; doi:10.1371/journal.pone.0029419)
Supplement: Table S4 — Base composition and strand bias in true bug mt genomes. (DOCX) [file pone.0029419.s004.docx]

**Table S4. Base composition and strand bias in true bug mt genomes.**

| **Species** | **A** | **T** | **A+T** | **AT-skew** | **C** | **G** | **C+G** | **GC-skew** |
| --- | --- | --- | --- | --- | --- | --- | --- | --- |
| *Aeschyntelus notatus* | 42.80 | 32.90 | 75.70 | 0.13 | 14.20 | 10.10 | 24.30 | -0.17 |
| *Aphelocheirus ellipsoideus* | 42.60 | 32.10 | 74.70 | 0.14 | 14.30 | 10.90 | 25.20 | -0.13 |
| *Coptosoma bifaria* | 40.90 | 30.40 | 71.30 | 0.15 | 16.20 | 12.50 | 28.70 | -0.13 |
| *Diplonychus rusticus* | 40.90 | 28.90 | 69.80 | 0.17 | 17.70 | 12.50 | 30.20 | -0.17 |
| *Dysdercus cingulatus* | 44.10 | 33.60 | 77.70 | 0.14 | 13.60 | 8.70 | 22.30 | -0.22 |
| *Enithares tibialis* | 44.40 | 31.70 | 76.10 | 0.17 | 14.30 | 9.60 | 23.90 | -0.20 |
| *Geocoris pallidipennis* | 43.30 | 32.50 | 75.80 | 0.14 | 13.90 | 10.30 | 24.20 | -0.15 |
| *Gerris* sp. | 43.90 | 31.80 | 75.70 | 0.16 | 14.40 | 10.00 | 24.40 | -0.18 |
| *Halyomorpha halys* | 43.10 | 33.30 | 76.40 | 0.13 | 13.60 | 10.10 | 23.70 | -0.15 |
| *Hydaropsis longirostris* | 39.00 | 36.20 | 75.20 | 0.04 | 14.40 | 10.40 | 24.80 | -0.16 |
| *Hydrometra* sp. | 43.90 | 34.70 | 78.60 | 0.12 | 12.30 | 9.00 | 21.30 | -0.15 |
| *Ilyocoris cimicoides* | 41.70 | 29.30 | 71.00 | 0.17 | 17.00 | 12.10 | 29.10 | -0.17 |
| *Laccotrephes robustus* | 43.00 | 27.60 | 70.60 | 0.22 | 18.20 | 11.20 | 29.40 | -0.24 |
| *Leptopus* sp. | 44.70 | 27.70 | 72.40 | 0.23 | 16.80 | 10.80 | 27.60 | -0.22 |
| *Lygus lineolaris* | 43.60 | 32.20 | 75.80 | 0.15 | 14.30 | 9.90 | 24.20 | -0.18 |
| *Macroscytus subaeneus* | 41.60 | 32.20 | 73.80 | 0.13 | 15.20 | 11.00 | 26.20 | -0.16 |
| *Malcus inconspicuus* | 44.30 | 33.50 | 77.80 | 0.14 | 13.30 | 8.90 | 22.20 | -0.20 |
| *Nerthra* sp. | 43.10 | 31.00 | 74.10 | 0.16 | 16.10 | 9.70 | 25.80 | -0.25 |
| *Neuroctenus parus* | 41.30 | 27.60 | 68.90 | 0.20 | 19.00 | 12.10 | 31.10 | -0.22 |
| *Nezara viridula* | 43.20 | 33.60 | 76.80 | 0.13 | 13.30 | 9.80 | 23.10 | -0.15 |
| *Ochterus marginatus* | 43.60 | 29.20 | 72.80 | 0.20 | 17.10 | 10.20 | 27.30 | -0.25 |
| *Orius niger* | 44.30 | 32.20 | 76.50 | 0.16 | 12.90 | 10.60 | 23.50 | -0.10 |
| *Paraplea frontalis* | 42.40 | 34.10 | 76.50 | 0.11 | 13.90 | 9.70 | 23.60 | -0.18 |
| *Phaenacantha marcida* | 44.30 | 29.20 | 73.50 | 0.21 | 15.80 | 10.70 | 26.50 | -0.19 |
| *Physopelta gutta* | 44.90 | 29.60 | 74.50 | 0.21 | 15.40 | 10.10 | 25.50 | -0.21 |
| *Riptortus pedestris* | 41.90 | 34.70 | 76.60 | 0.09 | 14.00 | 9.40 | 23.40 | -0.20 |
| *Saldula arsenjevi* | 43.20 | 31.40 | 74.60 | 0.16 | 14.50 | 10.90 | 25.40 | -0.14 |
| *Sigara septemlineata* | 41.30 | 33.80 | 75.10 | 0.10 | 14.30 | 10.50 | 24.80 | -0.15 |
| *Stenopirates* sp. | 43.90 | 38.60 | 82.50 | 0.06 | 10.00 | 7.50 | 17.50 | -0.14 |
| *Stictopleurus subviridis* | 42.50 | 33.20 | 75.70 | 0.12 | 14.10 | 10.20 | 24.30 | -0.16 |
| *Triatoma dimidiata* | 40.60 | 28.90 | 69.50 | 0.17 | 19.30 | 11.20 | 30.50 | -0.27 |
| *Valentia hoffmanni* | 41.40 | 32.40 | 73.80 | 0.12 | 15.90 | 10.30 | 26.20 | -0.21 |
| *Yemmalysus parallelus* | 41.50 | 34.90 | 76.40 | 0.09 | 12.30 | 11.30 | 23.60 | -0.04 |
| Avg. | 42.76 | 31.97 | 74.73 | 0.15 | 14.90 | 10.37 | 25.27 | -0.18 |
