# Supplementary material for: The Complete Mitochondrial Genome and Novel Gene Arrangement of the Unique-Headed Bug Stenopirates sp. (Hemiptera: Enicocephalidae)
Source: PLoS One. 2012 Jan 3;7(1):e29419. doi: 10.1371/journal.pone.0029419 (PMC3250431; doi:10.1371/journal.pone.0029419)
Supplement: Table S5 — Codon usage of protein-coding genes in the Stenopirates sp. mt genome. (DOCX) [file pone.0029419.s005.docx]

**Table S5. Codon usage of protein-coding genes in the *Stenopirates* sp. mt genome**

| **AA** | **Codon*** | **n** | **%** | **RSCU** | **AA** | **Codon** | **n** | **%** | **RSCU** |
| --- | --- | --- | --- | --- | --- | --- | --- | --- | --- |
| Ala (A) | GCU | 40 | 1.09 | 1.78 | Pro (P) | CCU | 43 | 1.17 | 1.45 |
|  | GCC | 5 | 0.14 | 0.22 |  | CCC | 2 | 0.05 | 0.07 |
|  | GCA | 44 | 1.20 | 1.96 |  | CCA | 73 | 1.99 | 2.45 |
|  | GCG | 1 | 0.03 | 0.04 |  | CCG | 1 | 0.03 | 0.03 |
| Cys (C) | UGU | 33 | 0.90 | 2 | Gln (Q) | CAA | 52 | 1.41 | 1.89 |
|  | UGC | 0 | 0.00 | 0 |  | CAG | 3 | 0.08 | 0.11 |
| Asp (D) | GAU | 49 | 1.33 | 1.92 | Arg (R) | CGU | 15 | 0.41 | 1.3 |
|  | GAC | 2 | 0.05 | 0.08 |  | CGC | 1 | 0.03 | 0.09 |
| Glu (E) | GAA | 73 | 1.99 | 1.95 |  | CGA | 28 | 0.76 | 2.43 |
|  | GAG | 2 | 0.05 | 0.05 |  | CGG | 2 | 0.05 | 0.17 |
| Phe (F) | UUU | 365 | 9.93 | 1.91 | Ser1 (S1) | AGU | 24 | 0.65 | 0.62 |
|  | UUC | 17 | 0.46 | 0.09 |  | AGC | 1 | 0.03 | 0.03 |
| Gly (G) | GGU | 55 | 1.50 | 1.36 |  | AGA | 91 | 2.48 | 2.35 |
|  | GGC | 3 | 0.08 | 0.07 |  | AGG | 2 | 0.05 | 0.05 |
|  | GGA | 92 | 2.50 | 2.27 | Ser2 (S2) | UCU | 87 | 2.37 | 2.25 |
|  | GGG | 12 | 0.33 | 0.3 |  | UCC | 5 | 0.14 | 0.13 |
| His (H) | CAU | 63 | 1.71 | 1.7 |  | UCA | 94 | 2.56 | 2.43 |
|  | CAC | 11 | 0.30 | 0.3 |  | UCG | 6 | 0.16 | 0.15 |
| Ile (I) | AUU | 436 | 11.86 | 1.91 | Thr (T) | ACU | 52 | 1.41 | 1.46 |
|  | AUC | 20 | 0.54 | 0.09 |  | ACC | 13 | 0.35 | 0.37 |
| Lys (K) | AAA | 116 | 3.16 | 1.89 |  | ACA | 77 | 2.10 | 2.17 |
|  | AAG | 7 | 0.19 | 0.11 |  | ACG | 0 | 0.00 | 0 |
| Leu1 (L1) | CUU | 14 | 0.38 | 0.16 | Val (V) | GUU | 32 | 0.87 | 1.38 |
|  | CUC | 0 | 0.00 | 0 |  | GUC | 2 | 0.05 | 0.09 |
|  | CUA | 18 | 0.49 | 0.21 |  | GUA | 58 | 1.58 | 2.49 |
|  | CUG | 0 | 0.00 | 0 |  | GUG | 1 | 0.03 | 0.04 |
| Leu2 (L2) | UUA | 469 | 12.76 | 5.45 | Trp (W) | UGA | 93 | 2.53 | 1.9 |
|  | UUG | 15 | 0.41 | 0.17 |  | UGG | 5 | 0.14 | 0.1 |
| Met (M) | AUA | 395 | 10.75 | 1.94 | Try (Y) | UAU | 165 | 4.49 | 1.86 |
|  | AUG | 13 | 0.35 | 0.06 |  | UAC | 12 | 0.33 | 0.14 |
| Asn (N) | AAU | 247 | 6.72 | 1.86 | Stop | UAA | 7 | 0.19 | 1.56 |
|  | AAC | 18 | 0.49 | 0.14 |  | UAG | 2 | 0.05 | 0.44 |

“*”: A total of 3675 codons from *Stenopirates* sp. are analyzed, excluding the start and stop codons. AA, amino acid; RSCU, Relative synonymous codon usage; n = frequency of each codon. % = n/3675.
