# Supplementary material for: The Complete Mitochondrial Genome and Novel Gene Arrangement of the Unique-Headed Bug Stenopirates sp. (Hemiptera: Enicocephalidae)
Source: PLoS One. 2012 Jan 3;7(1):e29419. doi: 10.1371/journal.pone.0029419 (PMC3250431; doi:10.1371/journal.pone.0029419)
Supplement: Table S6 — Different evolutionary patterns among protein-coding genes. (DOCX) [file pone.0029419.s006.docx]

**Table S6. Different evolutionary patterns among protein-coding genes**

| **PCGs** | **Ka** | **Ks** | **Ka/Ks** | **GC (%)** |
| --- | --- | --- | --- | --- |
| *atp6* | 0.26 | 0.92 | 0.28 | 17.47 |
| *atp8* | 0.49 | 0.73 | 0.67 | 9.62 |
| *cox1* | 0.11 | 1.02 | 0.10 | 26.06 |
| *cox2* | 0.21 | 0.99 | 0.21 | 18.98 |
| *cox3* | 0.17 | 0.97 | 0.17 | 23.09 |
| *cytb* | 0.16 | 1.04 | 0.15 | 21.72 |
| *nad1* | 0.22 | 0.70 | 0.31 | 18.06 |
| *nad2* | 0.42 | 0.79 | 0.54 | 10.64 |
| *nad3* | 0.30 | 0.91 | 0.33 | 15.54 |
| *nad4* | 0.34 | 0.67 | 0.51 | 15.86 |
| *nad4L* | 0.36 | 0.74 | 0.48 | 11.83 |
| *nad5* | 0.35 | 0.66 | 0.53 | 15.77 |
| *nad6* | 0.42 | 0.80 | 0.52 | 9.70 |
