# Supplementary material for: The Complete Mitochondrial Genome and Novel Gene Arrangement of the Unique-Headed Bug Stenopirates sp. (Hemiptera: Enicocephalidae)
Source: PLoS One. 2012 Jan 3;7(1):e29419. doi: 10.1371/journal.pone.0029419 (PMC3250431; doi:10.1371/journal.pone.0029419)
Supplement: Table S7 — Primer sequences used in this study. (DOCX) [file pone.0029419.s007.docx]

**Table S7: Primer sequences used in this study**

| **No. fragment^*^** | **Primer ID** | **Nucleotide sequence (5’-3’)** | **Reference** |
| --- | --- | --- | --- |
| 1 | TW- J1301 | GTTAAWTAAACTAATARCCTTCAAA | Simon *et al.*, 2006 |
|  | C1- N1738 | TTTATTCGTGGRAATGCYATRTC | Simon *et al.*, 2006 |
| 2 | C1-J1709 | AATTGGWGGWTTYGGAAAYTG | Simon *et al.*, 2006 |
|  | C1- N2776 | GGTAATCAGAGTATCGWCGNGG | Simon *et al.*, 2006 |
| 3 | C1-J2756 | ACATTTTTTCCTCAACATTT | Simon *et al.*, 2006 |
|  | C2-N3665 | CCACAAATTTCTGAACACTG | Simon *et al.*, 2006 |
| 4 | F-3665 | GATGCAATTCCTGGACGACTAAACCA | Present study |
|  | R-7795 | TCCATTTTCTTCTTGACTACCAGCAGC | Present study |
| 5 | N5-J7572 | AAAGGGAATTTGAGCTCTTTTWGT | Simon *et al.*, 2006 |
|  | N4-N8727 | AAATCTTTRATTGCTTATTCWTC | Simon *et al.*, 2006 |
| 6 | N4-J8641 | CCAGAAGAACACAAACCATG | Simon *et al.*, 2006 |
|  | N4L-N9629 | GTTTGTGAGGGTGCAATAGG | Simon *et al.*, 2006 |
| 7 | F-9629 | CAACATGAGCCTTGGGTAAC | Present study |
|  | R-9648 | ATTTTGCGAATTGGGTTATT | Present study |
| 8 | N4L-J9648 | TCCCAACACACCTTCACAAAC | Simon *et al.*, 2006 |
|  | CB- N11010 | TATCAACAGCAAATCCTCCTCA | Simon *et al.*, 2006 |
| 9 | CB-J10621 | CTCATACTGATGAAATTTTGGTTC | Simon *et al.*, 2006 |
|  | CB-N11526 | TTCTACTGGTCGTGCTCCAATTCA | Simon *et al.*, 2006 |
| 10 | F-11526 | TGCAATTTTACGATCCATTC | Present study |
|  | R-12888 | GTTACCTAAGGGATAACAGCGT | Present study |
| 11 | LR-J12888 | CCGGTCTGAACTCAGATCATGTA | Simon *et al.*, 2006 |
|  | LR-N13889 | ATTTATTGTACCTTTTGTATCAG | Simon *et al.*, 2006 |
| 12 | LR-J13342 | CCTTAGCACAGTTAAAATACTGC | Simon *et al.*, 2006 |
|  | LR-N14220 | TTATGCACATATCGCCCGTC | Simon *et al.*, 2006 |
| 13 | LR-J14197 | GTAAAYCTACTTTGTTACGACTT | Simon *et al.*, 2006 |
|  | SR-N14745 | GTGCCAGCAAYCGCGGTTATAC | Simon *et al.*, 2006 |
| 14 | SR- J14610 | ATAATAGGGTATCTAATCCTAGT | Simon *et al.*, 2006 |
|  | TM- N200 | ACCTTTATAARTGGGGTATGARCC | Simon *et al.*, 2006 |
| 15 | F-200 | AGATGCCTGATTAAAGGATTA | Present study |
|  | R-1301 | AAGATGGCTGAGTAAGGTTAT | Present study |

“*”: The orientation is shown in Figure 1.
